# Tailoring Physical and Sensory Properties of Tofu by the Addition of Jet-Milled, Superfine, Defatted Soybean Flour

**DOI:** 10.3390/foods8120617

**Published:** 2019-11-25

**Authors:** Ye-Na Kim, Syahrizal Muttakin, Young-Min Jung, Tae-Yeong Heo, Dong-Un Lee

**Affiliations:** 1 Department of Food Science and Technology, Chung-Ang University, 17546 Anseong, Korea; kyn6874@naver.com (Y.-N.K.); smuttakin@gmail.com (S.M.); jym20023@hanmail.net (Y.-M.J.); xodud1485@naver.com (T.-Y.H.); 2 Indonesian Agency for Agricultural Research and Development (IAARD), 12540 Jakarta, Indonesia

**Keywords:** defatted soybean flour, jet mill, super-fine powder, tofu, quantitative descriptive analysis, texture profile analysis

## Abstract

The use of defatted soybean flour (DSF) in food as a source of dietary fiber has been limited due to its rough texture and bitter taste. Our previous work indicates that superfine DSF prepared by jet milling could overcome these problems, as it positively affected physical and sensory properties. Therefore, differently sized DSFs were incorporated in tofu, and their impacts on physical and sensory properties were investigated in this study. Coarse DSF (Dv_50_ = 341.0 µm), fine DSF (Dv_50_ = 105.3 µm), and superfine DSF (Dv_50_ = 5.1 µm) were prepared by conventional sifting and jet milling. Tofu was made with a 5% addition of differently sized DSFs and without DSF (control tofu). The quality of tofu was evaluated by scanning electron microscopy, color measurement, texture profile analysis, and quantitative descriptive analysis. The tofu made with coarse and fine DSF showed negative changes in its physical and organoleptic qualities, such as reduced yields, a less pure color, a harder texture, and a rougher mouthfeel. However, the tofu made with superfine DSF showed only minimal changes in its qualities compared to the control. Therefore, superfine DSF is a promising fiber supplement that does not change the physical and sensory properties in the making of high-quality tofu.

## 1. Introduction

Tofu is a well-known Asian food made by coagulating soymilk and then pressing the resulting curds into soft white blocks. The coagulation of soymilk is done with coagulants such as CaSO_4_, CaCl_2_, or glucono-δ-lactone (GDL). Transglutaminase, which can catalyze the cross-linking reactions between soy proteins, is also being investigated as a coagulant for tofu [1]. During the process of making tofu, soymilk is heated to 90 °C. Thermal treatment is routinely used in the production of tofu to dissociate, denature, and aggregate the soy protein, inhibit microbial growth, reduce the beany flavor, and inactivate undesirable biological compounds such as trypsin inhibitors and lipoxygenase [2,3].

Tofu is a good source of protein. Its general composition is 84.6% water, 8.1% protein, 4.8% lipids, and 1.9% carbohydrates [4]. As it is derived from soybeans, tofu is also rich in phenolic compounds, such as isoflavones, which have antioxidant activities and health benefits [5,6,7]. Although tofu contains valuable nutritional compounds, it has little dietary fiber because the soybean pulp is removed during production. Aside from the health benefits of dietary fiber, consumer demand for unrefined or minimally processed whole foods is increasing. The small amount of dietary fiber in tofu and increasing consumer demand for whole foods could be resolved if a fiber-enriched tofu could be produced with dietary fiber from unpolished beans.

Significant effort has been made to obtain dietary fiber from vegetable products as a functional ingredient. Examples include baked foods with added dietary fiber [8,9], fiber-enriched cocoa powder [10], dietary fiber-enriched biscuits using okra flour [11], whole wheat/soy flour bread [12], and breads containing corn bran [13]. However, there has been little research to verify the effects of the addition of dietary fiber to tofu.

One of the dietary ingredients derived from soybeans is defatted soybean flour (DSF). DSF contains high levels of dietary fiber, along with proteins, carbohydrates, and fats [14]. Although DSF is produced in large quantities as a by-product of soybean oil, the addition of DSF to food as a source of fiber has been restricted due to its rough texture and bitter taste.

We have previously shown that superfine DSF powder could be prepared by conventional milling and sifting followed by jet milling [15]. Jet-milled DSF showed significant reductions in bitterness and roughness by descriptive analysis with trained panelists, and its water-holding capacity, water-solubility index, and swelling capacity were significantly increased. These results indicate the possibility of using superfine DSF as a functional ingredient to modify the physical properties of food without any negative changes to its sensory properties. The objective of this study was to apply jet-milled, superfine DSF powder to the preparation of fiber-enriched tofu and to verify its effects on the physical and sensory properties of tofu.

## 2. Materials and Methods

### 2.1. Materials

Soybeans (*Glycine max* Merr.) were obtained from a local market (NH market, Anseong, Korea). Food-grade DSF was obtained from Sam Chang Industry Co. (Anseong, Korea). Magnesium chloride, glucono-δ-lactone, and sodium chloride were obtained from Samchun Chemical Co. (Seoul, Korea). All of those chemicals were food grade.

### 2.2. Preparation of Coarse, Fine, and Superfine Fractions of DSF

**Figure 1:**
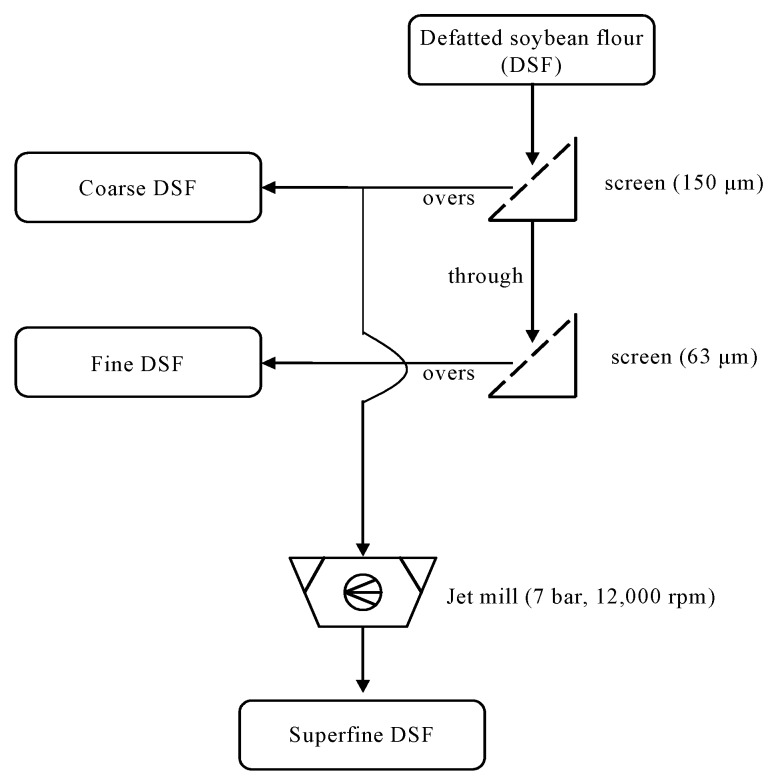
The process flow for the preparations of coarse, fine, and superfine defatted soybean flour (DSF) using serial screening and jet milling.

The DSF powder was sieved twice (150 and 63 μm testing sieves; Nonaka Rikaki, Tokyo, Japan) to obtain coarse and fine fractions (Figure 1). The powders that could not pass through the 150 μm sieve were collected as the coarse fraction. The powders that passed through the 150 μm sieve but were retained by the 63 μm sieve were collected as the fine fraction. The coarse fraction of DSF was then further pulverized by a fluidized-bed jet mill (CGS-10, Netzsch GmbH, Selb, Germany), yielding a superfine fraction. Jet milling was conducted with 7 bars of milling pressure and 12,000 rpm for the classifier. The particle size distributions of the DSFs were determined using a laser diffraction particle sizer (Mastersizer 3000, Malvern Instruments, Malvern, UK).

### 2.3. Tofu Preparation

Tofu was prepared as described with some modifications [16,17]. Soybeans (9 kg) were soaked in tap water with a soybean-to-water ratio of 1:3 (w/w) for 12 h at room temperature. The swollen soybeans were drained and poured into a soymilk grinder equipped with a soy pulp separator (JH3211A, Junghoon Co., Seoul, Korea) using distilled water (1:8, w/w). The resulting soymilk was heated to 95 °C for 5 min. The hot soymilk was then poured into a stainless steel container, and the respective DSFs (coarse, fine, and superfine DSF) were added gradually to the soymilk to final concentrations of 5%. The final volume of soymilk was 28.8 L. The soymilk with DSF was cooled to 80 °C and combined with coagulant solution. The coagulant solution was composed of 45 g of magnesium chloride, 74 g of GDL, and 72 g of sodium chloride in 1 L of distilled water. The curd was left at ambient temperature to coagulate for 10 min before being transferred to a perforated stainless steel container lined with cheesecloth. The whey in the curd was removed by pressing at 0.2 kg/cm^2^ for 25 min. The tofu yield was expressed as the kg of tofu per 28.8 L of soymilk. Ordinary tofu without any addition of DSF was used as a control. The fibrous tofu varieties containing 5% coarse, fine, and superfine DSFs were labeled as tofu with coarse DSF, tofu with fine DSF, and tofu with superfine DSF, respectively.

### 2.4. Moisture Determination

The amount of moisture in each tofu sample was determined according to the AACC method [18] with some modifications. A sliced, 1.0 g amount of freshly prepared tofu was placed on an aluminum dish. The sample was then dried at 105 °C in a drying oven for 24 h. The moisture content was calculated as the percentage of water content per 1 g of fresh tofu.

### 2.5. Microstructure Observation

The tofu sample for scanning electron microscopy (SEM) was cut into cubes (5 × 5 × 5 mm), and freeze-dried. Lyophilized samples were coated with platinum–lead (Pt–Pb) by an ion coater (E-1010, Eiko Co., Hyogo, Japan) and observed under a SEM system (S-3400N, Hitachi, Tokyo, Japan).

### 2.6. Color Measurement

The color of the tofu was determined based on the CIE L* (lightness), a* (redness/greenness), and b* (yellowness/blueness) values using a colorimeter (UltraScan Pro, HunterLab, Reston, VA, USA). A standard white plate with L* = 97.49, a* = 0.13, and b* = 0.04 was used for calibration. The tofu samples were cut into 5.0 × 5.0 × 1.0 cm cubes, and their colors were measured. Each sample was measured three times. The color differentiation (Δ*E*) between the control tofu and tofu containing 5% DSF was calculated as follows:

$$\Delta E=\sqrt{{\left(\Delta L^{*}\right)}^{2}+{\left(\Delta a^{*}\right)}^{2}+{\left(\Delta b^{*}\right)}^{2}}$$

### 2.7. Texture Profile Analysis (TPA)

The texture of the tofu was analyzed according to a texture profile analysis (TPA) using a texture analyzer (TA-XT2i, Stable Micro Systems, Surrey, UK) with a 35 mm diameter compression plunger. Each tofu sample was cut into a cylindrical shape with a diameter of 20 mm and a height of 15 mm. Each cylindrical sample was placed on the center of the TPA plate and compressed twice to 50% of its original height by a cylinder probe (20 mm in diameter, P/20) at a constant speed of 2 mm/s. The texture profile analysis curve was recorded, and the hardness, adhesiveness, springiness, cohesiveness, gumminess, and chewiness were calculated automatically [19].

### 2.8. Sensory Evaluation Using Descriptive Analysis 

The sensory properties of the tofu samples were evaluated by the descriptive analysis method [20] with some modifications made to use standard reference products available in local markets. For the sensory evaluation, freshly made tofu was cut into 3.0 cm cubes and placed in white dishes with three-digit random numbers. The evaluation was repeated three times for each panelist (*n* = 3 × 15). The samples were presented monadically to the judges at room temperature under white fluorescent lighting. Fifteen panelists, consisting of Chung-Ang University graduate students who were experienced in descriptive analysis, were first trained with previously identified standard references (Table 1). Four sensory attributes (hardness, springiness, mouthfeel, and beany flavor) were adapted and slightly modified from previous studies [21]. The trained panelists were asked to score the sensory attributes of the tofu samples. Before and between the tests, the panelists were instructed to drink water to clean their mouths. A nine point intensity scale was used to express the intensity of each sensory attribute.

### 2.9. Statistical Analysis of the Data

All experiments were performed in triplicate. The results are expressed as the means ± standard deviations. ANOVA and Duncan’s multiple range comparison tests were performed using the SPSS version 20.0 software (IBM Corp., Armonk, NY, USA) for statistical analyses; *p*-values < 0.05 were considered significant.

## 3. Results and Discussion

### 3.1. Particle Size Distribution of Defatted Soy Flour

The particle size distributions in the DSF samples are shown in Table 2. The results for each are presented as the mean of the volume-weighted diameter (D_[4, 3]_), the equivalent diameter at a cumulative volume of 10% (Dv_10_), the equivalent diameter at a cumulative volume of 50% (Dv_50_), the equivalent diameter at a cumulative volume of 90% (Dv_90_), and the homogeneity (span value). The span value shows the uniformity of the particle size. A smaller span value indicates a more homogenous size and a narrower particle-size distribution. All DSF samples had low span values below 1.50.

### 3.2. Yield and Moisture Content of Tofu

The yields and moisture contents of the tofu samples made with the different DSFs are presented in Table 3. The yield of tofu increased slightly with a reduction in DSF particle size. There were no significant changes in the tofu yield between the control tofu (without DSF) and the tofu samples with coarse, fine, and superfine DSF. The moisture content of the tofu was increased significantly by the application of jet milling to the DSF. The moisture contents of the tofu with coarse and fine DSF were almost identical (69.9% and 69.8%, respectively). However, the moisture content of the tofu made with superfine DSF was 75.6%, which is similar to that of the control tofu.

A previous report indicated that the yield of tofu is strongly related to the aggregation of soy protein, which traps water in the tofu structure [22]. However, an increased solid content seems to have a negative effect on the moisture content of tofu. Cai et al. [23] showed that tofu containing a high proportion of solids had a low moisture content. The same result, that the interaction of the components of soybeans and additional solid particles decreased the water holding capacity of tofu, was also reported by Lim et al. [24]. These results suggest that the addition of DSF to tofu could reduce its ability to retain water and form a gel and might result in tofu with a reduced moisture content. In this study, the addition of coarse and fine DSF to tofu resulted in a moisture content of 70%, whereas the moisture content of tofu with superfine DSF was 75.6%. The increased moisture content of tofu with superfine DSF can be explained by the increased hydration properties of the superfine DSF. Our previous studies have shown that superfine DSF has increased hydration properties, such as a water-holding capacity and swelling capacity [15]. The water-holding capacity and swelling capacity explain the ability of a moist material to retain water. The higher water-holding capacity of the jet-milled, superfine DSF may explain the increased moisture content and yield of the tofu samples.

### 3.3. Microstructure and Color of Tofu

**Figure 2:**
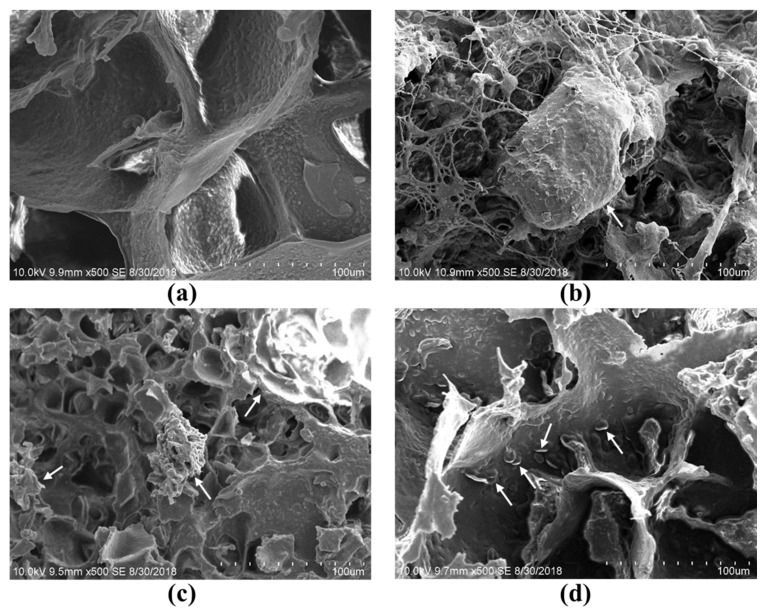
Scanning electron micrographs of tofu made with different fibers at 500× magnification: (**a**) tofu made without DSF, (**b**) tofu made with coarse DSF, (**c**) tofu made with fine DSF, and (**d**) tofu made with superfine DSF. The arrows represent DSFs.

Figure 2 shows scanning electron micrographs of the tofu with different DSFs. As shown in Figure 2a, tofu made without DSF has an obvious gel network and forms a large cell-like structure with a flat wall. This gel network seems to be affected by the addition of DSFs in the other samples. Tofu made with the coarse DSF (Figure 2b) had a non-uniform structure, and a flat wall was not found. Tofu made with fine DSF (Figure 2c) showed a denser network than the control. The microstructure of the tofu made with the superfine DSF (Figure 2d) was more similar to the control than the tofu made with coarse and fine DSF. The DSFs in the tofu made with superfine DSF appear to be entrapped inside the wall structure. Ullah et al. [25] also found a change in the tofu matrix after the addition of Okara dietary fiber due to the interaction of fiber with proteins.

Color is an important property of tofu. Good-quality tofu should have a light-yellow or white color. Table 3 shows the effect of DSF particle size on tofu’s color. The lightness (L* value) of the control tofu was 87.55 ± 0.24 and decreased with the addition of coarse and fine DSFs. The darkening of tofu by the addition of fibrous material was reported by Murugkar [26], who reported that an increased particle size of ground sprout seed in soymilk resulted in a decreased whiteness index of tofu products. However, there was no significant difference in the lightness between the control and the tofu with superfine DSF.

The same trends were observed for the a* and b* values of the tofu. The addition of coarse and fine DSFs resulted in increased a* and b* values, but the addition of superfine DSF did not change the a* and b* values. The tofu with coarse and fine DSFs had a greater Δ*E* value (meaning that the color of these samples differed significantly from the control tofu; *p* < 0.05). These results are in accordance with previous reports, showing that, compared to raw materials, jet-milled, superfine powders produce brighter and more neutral colors [15,17].

### 3.4. Textural Properties of Tofu

The textural properties of tofu are associated with its physical properties and play an important role in consumer acceptability [27]. Table 4 shows the results of a texture profile analysis of the control and tofu with DSFs. The addition of larger particles of DSF (coarse and fine DSFs) increased the hardness of the tofu significantly to 0.61 ± 0.05 and 0.60 ± 0.06, respectively, whereas the addition of superfine DSF resulted in no change in hardness compared to the control tofu. The same trend was observed in the springiness and cohesiveness of the tofu. The addition of coarse and fine DSFs reduced the springiness and cohesiveness of the tofu, but the addition of superfine DSF did not change the springiness and cohesiveness of the control tofu. However, DSF’s addition did not change the chewiness and gumminess for any of the tofu samples.

Hardness was measured by the absolute peak force on the first down stroke during TPA analysis. The addition of conventional DSF to soymilk resulted in an increase in the solid content of the tofu product. Moreover, the force required to break down the fibrous tofu structure was higher than for normal tofu. In contrast, the jet-milled DSF did not behave as a solid in the tofu product due to its superfine particle size. The hardness value of tofu is also related to its moisture content [28]. As described previously, the tofu with superfine DSF had as high a moisture content as the control tofu. These properties led to a soft-textured tofu with superfine DSF. Springiness is a measure of how well a product physically springs back after it has been deformed during the first compression, and products with higher springiness are thought to have higher elasticity. The control tofu and tofu made with superfine DSF had almost identical springiness values of 0.94 ± 0.02 and 0.95 ± 0.02, respectively, whereas the tofu made with coarse and fine DSFs showed slightly reduced springiness values. There were no significant differences in chewiness and gumminess between the control tofu and the tofu with different DSFs.

### 3.5. Sensory Analysis of Tofu by Descriptive Analysis

The sensory properties of the control and DSF tofu samples were analyzed by the descriptive analysis method (Table 5). Four sensory attributes (hardness, springiness, mouthfeel, and beany flavor) with the previously identified standards are shown in Table 1. The addition of 5% coarse DSF resulted in significant increases in hardness, mouthfeel, and beany flavor compared to the control tofu. The increases of hardness and mouthfeel are negative results, since consumers expect tofu with a soft and smooth structure. The increase of beany flavor can be good or bad depending on individual preference. There was no significant change in springiness.

The increased hardness in tofu by the addition of 5% DSF was relieved by the reduction of DSF’s particle size. The hardness of the tofu with coarse DSF was 6.0 ± 1.2, while the hardnesses of the tofu with fine and superfine DSFs were 5.1 ± 1.3 and 3.9 ± 0.9, respectively. The mouthfeel showed a similar tendency. The addition of larger particles of DSF (coarse and fine DSFs) resulted in tofu with a rougher mouthfeel. However, the addition of superfine DSF had a reduced mouthfeel, which indicates that soft structured tofu was made due to the smaller particle size of the DSF. A smaller particle size resulted in a less beany flavor compared to the larger DSF. The control tofu had a beany flavor score of 3.9 ± 1.1. The addition of coarse DSF increased the beany flavor score to 6.4 ± 1.2. The addition of superfine DSF resulted in a beany flavor score of 4.7 ± 1.1, which is an increase in value but is not significantly different from the score of the control tofu (*p* < 0.05).

The descriptive analysis results showed the same trend as the textural property values obtained using the texture analyzer, especially for hardness and springiness. The mouthfeel attribute was defined as a rough particle size texture when panelists tasted the tofu. As expected, a larger particle size increased the mouthfeel value of the tofu. The same result was found by Kim et al. [29], who reported that the addition of shell powder during tofu processing increased the mouthfeel score. The limited increase in beany flavor of the tofu with superfine DSF was unexpected. One possible explanation is that compounds inducing a beany flavor are removed during sieving or jet milling. We previously showed that superfine DSF can be a food ingredient because it decreases bitter taste and increases the sweetness of DSF [15]. Our descriptive analysis results indicate that the addition of superfine DSF powder up to a value up to 5% does not change the sensory properties of tofu. Thus, superfine DSF could be a good raw material for enhancing the dietary fiber content of tofu products.

## 4. Conclusions

The results of this study show that fibrous tofu consist of up to 5% DSF. However, conventionally prepared DSFs with an average particle size larger than 100 μm negatively affected the sensory properties of tofu. The addition of such DSFs resulted in a lower yield, lower moisture content, harder texture, rougher mouthfeel, and increased beany flavor compared to the control tofu. On the other hand, the tofu made with jet-milled DSF overcame all of these disadvantages. The color, textural properties, and sensory qualities were statistically identical to those of the tofu without DSF. Our descriptive analysis results show that consumers will accept tofu with 5% superfine DSF because even trained panelists could not distinguish it from control tofu due to the ultrafine characteristics of the superfine DSF. Therefore, it can be concluded that superfine DSF is a promising additive, for enriching fiber, that does not change the physical or sensory properties during the production of high-quality tofu.

## Figures and Tables

**Table 1:** Descriptors used for the sensory evaluation of tofu by descriptive analysis.

Descriptors	Definition		Position	
			1	9
Hardness	Force required to compress the sample with molars	Intensity	Soft	Hard
		Standard	Cream cheese	Raisin
Springiness	Degree to which sample returns to the original shape after compression with molars	Intensity	Not springy	Very springy
		Standard	Cream cheese	Chewy cheese
Mouthfeel	Perception of the particles against the roof of the mouth	Intensity	Smooth	Rough
		Standard	Soft curd	Defatted rice bran
Beany flavor	Flavor associated with soy foods	Intensity	No bean flavor	Strong bean flavor
		Standard	Soymilk, 50%	Soymilk, 99%

**Table 2:** Particle size distributions of the defatted soybean flours (DSFs).

Sample	D_[4, 3]_	Dv_10_	Dv_50_	Dv_90_	Span
Coarse DSF	344.0 ± 3.0 ^a^	154.3 ± 1.5 ^a^	341.0 ± 2.0 ^a^	546.3 ± 6.0 ^a^	1.15 ± 0.01
Fine DSF	106.3 ± 0.6 ^b^	64.03 ± 0.4 ^b^	105.3 ± 0.6 ^b^	158.0 ± 1.0 ^b^	0.89 ± 0.01
Superfine DSF	5.7 ± 0.1 ^c^	2.4 ± 0.1 ^c^	5.1 ± 0.1 ^c^	9.7 ± 0.1 ^c^	1.44 ± 0.00

Values are the means of triplicates and expressed as the means ± standard deviations. No significant difference was observed between the means designated by the same letter (Duncan’s *p* < 0.05).

**Table 3:** The effect of the DSF particle size on the yield, moisture content, and color of the tofu.

Sample	Yield (%)	Moisture (%)	Tofu Color			
			L*	a*	b*	Δ*E*
Tofu without DSF	50.1 ± 4.4 ^ab^	75.8 ± 1.1 ^a^	87.55 ± 0.24 ^a^	0.32 ± 0.07 ^b^	14.22 ± 0.36 ^b^	-
Tofu with coarse DSF	47.5 ± 2.5 ^b^	69.9 ± 2.0 ^b^	86.16 ± 0.57 ^b^	0.87 ± 0.45 ^a^	14.67 ± 0.28 ^a^	1.67 ± 0.59 ^a^
Tofu with fine DSF	48.7 ± 3.5 ^ab^	69.8 ± 3.9 ^b^	86.11 ± 0.31 ^b^	0.75 ± 0.15 ^a^	14.94 ± 0.13 ^a^	1.69 ± 0.29 ^a^
Tofu with superfine DSF	51.0 ± 2.0 ^a^	75.6 ± 1.5 ^a^	87.10 ± 0.40 ^a^	0.50 ± 0.08 ^ab^	14.93 ± 0.36 ^ab^	0.88 ± 0.18 ^b^

Values are the means of triplicates and expressed as the means ± standard deviations. No significant difference was observed between the means designated by the same letter (Duncan’s *p* < 0.05).

**Table 4:** The effect of DSF particle size on the textural properties of tofu.

Sample	Textural Property				
	Hardness (*N*)	Springiness	Cohesiveness	Chewiness (*J*)	Gumminess (*N*)
Tofu without DSF	0.52 ± 0.05 ^b^	0.94 ± 0.02 ^ab^	0.60 ± 0.02 ^ab^	0.29 ± 0.04 ^a^	0.31 ± 0.04 ^a^
Tofu with coarse DSF	0.61 ± 0.05 ^a^	0.90 ± 0.02 ^c^	0.58 ± 0.03 ^b^	0.32 ± 0.03 ^a^	0.35 ± 0.03 ^a^
Tofu with fine DSF	0.60 ± 0.06 ^a^	0.92 ± 0.02 ^bc^	0.59 ± 0.02 ^b^	0.32 ± 0.03 ^a^	0.33 ± 0.05 ^a^
Tofu with superfine DSF	0.53 ± 0.07 ^b^	0.95 ± 0.02 ^a^	0.62 ± 0.03 ^a^	0.32 ± 0.05 ^a^	0.32 ± 0.04 ^a^

Values are the means of triplicates and are expressed as the means ± standard deviations. No significant difference was observed between the means designated by the same letter (Duncan’s *p* < 0.05).

**Table 5:** The effect of DSF particle size on the sensory properties of tofu.

Sample	Hardness	Springiness	Mouthfeel	Beany Flavor
Tofu without DSF	3.7 ± 1.6 ^b^	5.0 ± 1.4 ^a^	2.9 ± 0.7 ^b^	3.9 ± 1.1 ^c^
Tofu with coarse DSF	6.0 ± 1.2 ^a^	4.6 ± 1.4 ^a^	5.9 ± 1.1 ^a^	6.4 ± 1.2 ^a^
Tofu with fine DSF	5.1 ± 1.3 ^a^	4.7 ± 1.2 ^a^	5.6 ± 1.0 ^a^	5.3 ± 1.5 ^ab^
Tofu with superfine DSF	3.9 ± 0.9 ^b^	5.2 ± 1.2 ^a^	3.5 ± 0.7 ^b^	4.7 ± 1.1 ^bc^

Values are means of three replicates and 15 judges (*n* = 3 × 15) and are expressed as the means ± standard deviations. No significant difference was observed between the means designated by the same letter (Duncan’s *p* < 0.05).

## References

[1] Tang C.-H. (2007). Effect of thermal pretreatment of raw soymilk on the gel strength and microstructure of tofu induced by microbial transglutaminase. LWT-Food Sci. Technol..

[2] Kumar V., Rani A., Tindwani C., Jain M. (2003). Lipoxygenase isozymes and trypsin inhibitor activities in soybean as influenced by growing location. Food Chem..

[3] Liu H.-H., Chien J.-T., Kuo M.-I. (2013). Ultra high pressure homogenized soy flour for tofu making. Food Hydrocoll..

[4] USDA National Nutrient Database for Standard Reference. https://ndb.nal.usda.gov/ndb/foods/show/16427.

[5] Rekha C.R., Vijayalakshmi G. (2013). Influence of processing parameters on the quality of soycurd (tofu). J. Food Sci. Technol..

[6] Ishihara M., Singh H., Chung G., Tam C. (2007). Content composition and antioxidant activity of isoflavones in commercial and homemade soymilk and tofu. J. Sci. Food Agric..

[7] Hui E., Henning S.M., Park N., Heber D., Liang V., Go W. (2001). Genistein and Daidzein/Glycitein Content in Tofu. J. Food. Compos. Anal..

[8] Protonotariou S., Batzaki C., Yanniotis S., Mandala I. (2016). Effect of jet milled whole wheat flour in biscuits properties. LWT-Food Sci. Technol..

[9] Ktenioudaki A., Gallagher E. (2012). Recent advances in the development of high-fibre baked products. Trends Food Sci. Technol..

[10] Lecumberri E., Mateos R., Izquierdo-Pulido M., Rupérez P., Goya L., Bravo L. (2007). Dietary fibre composition, antioxidant capacity and physico-chemical properties of a fibre-rich product from cocoa (Theobroma cacao L.). Food Chem..

[11] Grizotto R.K., Rufi C.R.G., Yamada E.A., Vicente E. (2010). Evaluation of the quality of a molded sweet biscuit enriched with okara flour. Ciência Tecnol. Aliment..

[12] Shogren R.I., Mohamed A.A., Carriere C.J. (2003). Sensory analysis of whole wheat/soy flour breads. J. Food Sci..

[13] Singh M., Liu S.X., Vaughn S.F. (2012). Effect of corn bran as dietary fiber addition on baking and sensory quality. Biocatal. Agric. Biotechnol..

[14] Berk Z. (1992). Technology of Production of Edible Flours and Protein Products from Soybeans.

[15] Muttakin S., Kim M.S., Lee D.-U. (2015). Tailoring physicochemical and sensorial properties of defatted soybean flour using jet-milling technology. Food Chem..

[16] Lee C.-Y., Kuo M.-I. (2011). Effect of γ-polyglutamate on the rheological properties and microstructure of tofu. Food Hydrocoll..

[17] Phat C., Li H., Lee D.U., Moon B., Yoo Y.B., Lee C. (2015). Characterization of Hericium erinaceum powders prepared by conventional roll milling and jet milling. J. Food Eng..

[18] Eagen M.N., AACC (1983). Approved Methods of the American Association of Cereal Chemists.

[19] Bourne M.C. (2002). Food Texture and Viscosity: Concept and Measurement.

[20] Kamizake N.K.K., Silva L.C.P., Prudencio S.H. (2018). Impact of soybean aging conditions on tofu sensory characteristics and acceptance. J. Sci. Food Agric..

[21] Ziegler G.R., Mongia G., Hollender R. (2001). The role of particle size distribution of suspended solids in defining the sensory properties of milk chocolate. Int. J. Food Prop..

[22] Prabhakaran M.P., Perera C.O., Valiyaveettil S. (2006). Effect of differen coagulants on the isoflavone levels and physical properties of prepared firm tofu from South East Asia. Int. J. Food Prop..

[23] Cai T.D., Chang K.C., Shih M.C., Hou H.J., Ji M. (1997). Comparison of bench and production scale methods for making soymilk and tofu from 13 soybean varieties. Food Res. Int..

[24] Lim B.T., deMan J.M., deMan L. (1990). Yield and quality of tofu made from soybeans and soypeanut blends. J. Am. Oil Chem. Soc..

[25] Ullah I., Hu Y., You J., Yin T., Xiong S., Din Z.-U., Huang Q., Liu R. (2019). Influence of okara dietary fiber with varying particle sizes on gelling properties, water state and microstructure of tofu gel. Food Hydrocoll..

[26] Murugkar D.A. (2014). Effect of different process parameters on the quality of soymilk and tofu from sprouted soybean. J. Food Sci. Technol..

[27] Lee Y.C., Rosenau J.R., Peleg M. (1983). Rheological characteristic of tofu. J. Texture Stud..

[28] Prabhakaran M.P., Perera C.O., Valiyaveettil S. (2005). Quantification of Isoflavones in Soymilk and Tofu from South East Asia. Int. J. Food Prop..

[29] Kim Y.S., Choi Y.M., Noh D.O., Cho S.Y., Suh H.J. (2007). The effect of oyster shell powder on the extension of the shelf life of tofu. Food Chem..

